# An Eye on COVID: Hurricane Preparedness at a COVID-19 Alternative Care Site

**DOI:** 10.1017/dmp.2020.318

**Published:** 2020-09-03

**Authors:** Meghan Maslanka, Jacob A. Hurwitz

**Affiliations:** Louisiana Department of Health Medical Monitoring Station, LSU, Emergency Medicine and EMS and Disaster Medicine, New Orleans, Louisiana; Louisiana Department of Health Medical Monitoring Station, Tulane University School of Medicine, School of Public Health and Tropical Medicine, New Orleans, Louisiana

**Keywords:** alternative care site, COVID-19, hurricane

## Abstract

**Background:**

In March 2020, the Louisiana Department of Health activated the Medical Monitoring Station (MMS) in downtown New Orleans. This alternative care site is designed to decompress hospitals and nursing homes overwhelmed by the coronavirus disease 2019 (COVID-19) pandemic. Given the city’s historic vulnerability to hurricanes, planning for possible tropical weather events has been a priority for MMS leadership.

**Methods:**

The planning process incorporated input from all sectors/agencies working at the facility, to ensure consistency and cohesion. The MMS Shelter-in-Place Plan (MSIPP) was created, and a comprehensive tabletop exercise was conducted.

**Results:**

Six planning topics emerged as a result of the planning process and were used to create a comprehensive plan for sheltering-in-place. These topics address hurricane preparedness for patient care, interfacility coordination, wrap-around services, medical logistics, essential staffing, and incident command during a shelter-in-place scenario.

**Conclusions:**

The MSIPP created by the MMS helped to maximize patient safety and continuity of operations during a real-world event. Select pieces of the plan were activated to meet the needs and threat level of Tropical Storm Cristobal. This experience reinforced the need for originality, scalability, and flexibility in building emergency operations plans in the midst of an unprecedented pandemic.

When New Orleans emerged as a coronavirus disease 2019 (COVID-19) hotspot in April 2020, the state opened multiple alternative care sites to decompress health-care facilities overwhelmed with COVID-19 patients. The largest of these sites is the Medical Monitoring Station (MMS), located at the Ernest N. Morial Convention Center (MCCNO). The MMS is a facility for COVID-19 positive patients who require medical monitoring and/or a facility in which to isolate. The MMS requires a referral from a health-care provider and accepts patients for transfer from other health-care settings. Acute-care hospitals, emergency departments, urgent cares, clinics, behavioral health facilities, and nursing homes are able to refer patients to the facility, which is equipped to provide basic medical care, similar to that of a medical-surgical unit of a hospital. Critical care staff, ventilators, laboratory services, and imaging are not available. Patients who deteriorate or experience medical emergencies are stabilized and transported to definitive care by onsite Emergency Medical Services (EMS).

Although the initially steep rate of COVID-19 cases in Louisiana has plateaued, the MMS remains operational as the state moves toward reopening, standing ready to accommodate a no-notice resurgence in cases. However, sustaining operations into hurricane season requires that the facility itself be well-prepared for tropical weather events. Since Hurricane Katrina, Louisiana’s coastal communities, New Orleans included, have developed robust plans for evacuation ahead of a major hurricane.^1^ However, COVID-19 is expected to complicate this practice; it is anticipated that out-of-state sheltering may be inaccessible, given New Orleans’s highly publicized COVID-19 challenges and the evolving travel restrictions implemented across Southern states. Meanwhile, evacuation transportation plans are not designed for the COVID-19 pandemic. A significant increase in the quantity of vehicles would be required to evacuate citizens in a socially distant manner. The ability to shelter citizens is also impacted by the need to reduce viral spread. Shelters must prepare to implement strict infection control measures and reduce the impact of unidentified COVID-19 carriers as much as possible. These amended shelter guidelines^2^ greatly reduce the capacity of previously identified evacuation shelters. Louisiana’s low-lying nursing homes also plan to evacuate patients and staff ahead of storms, often directly to a partnering facility. Numerous receiving nursing homes have rescinded these agreements given their impacts during the COVID-19 emergency, leaving flood-prone facilities scrambling for alternatives as hurricane season begins.

The MMS will likely assist with sheltering COVID-19 positive individuals during a hurricane, as COVID-19 positive individuals who cannot be safely isolated at receiving nursing homes or shelters can be well accommodated at the MMS. This will reduce the stress placed on hospitals that may otherwise be asked to shelter these patients through a storm. The MMS will remain focused on its mission as a medical facility during a hurricane and does not plan to provide shelter to the general population without a referral from a health-care provider. Given the risk of public utility and transportation disruption during a hurricane, MMS leadership drafted a detailed MMS Shelter-In-Place Plan (MSIPP) to ensure the MMS’s ability to continue safe and effective operations during a storm.

## PLANNING PROCESS

With the overarching goal of sheltering-in-place, MMS leadership drafted a plan outlining high-level objectives for each service required to sustain patient care and staff safety. Concurrent with the drafting of the MSIPP, MMS leadership assigned relevant objectives to specific partners and sections already involved in MMS operations. From each sector, MMS leadership requested the drafting of an annex detailing tactical pre- and poststorm actions and any additional resources required to fulfill their assigned responsibilities. A total of 20 annexes were incorporated into the MSIPP. Table 1 identifies key participants, their respective planning objectives, and their operational roles.

**TABLE 1:** Summary of MSIPP^a^ Sectors and Associated Planning Objectives

Sector	Operational Role	Planning Objectives
Medical Operations	Direct patient care; medical direction; discharge planning; case management; COVID-19 testing; PPE^b^ guidance	Reverse triage; delivery of critical care for decompensating patients; continuity of patient care; COVID-19 testing
Pharmacy Support	Pharmaceutical supply and management	Sustainment of pharmaceutical supply and distribution
Emergency Medical Services	Patient transportation from transferring facility to MMS^c^; emergency medical transport to hospital for decompensating patients	Establishment of safe transportation limits during storm; continuity of access to MMS and external healthcare facilities
Logistics	Medical and non-medical item inventory management; warehouse operations; distribution of supplies within MMS; PPE management; medical oxygen supply	Sustainment of medical, non-medical, and PPE inventory; continuity of warehouse management and materials movement within MMS
Tactical Communications & IT Support	Internal and external tactical communications; IT^d^ support	Continuity of radio, phone, and internet communications
Louisiana State Police	Security of MMS, ICP^e^, and surrounding areas	Continuity of security operations; potential for increased security concerns post-storm
Wrap-Around Services	Patient and staff feeding; medical and non-medical custodial services; medical linens and laundry; medical and non-medical waste management	Continuity of feeding, including special medical diets; sustainment of linen supply; post-storm waste management capacity
Louisiana National Guard	Military logistics support; access control	Contingency planning for provision of electrical generators in the event of loss of municipal power supply
Site Safety & Fire Safety	Staff and patient safety; fire suppression and safety of MMS	Pre- and post-storm staff safety; continuity of onsite firefighting capabilities; post-storm damage assessments
Public Information	Media relations	Continuity of response to media inquiries; public messaging concerning facility use and safety
Finance Section	Resource procurement and tracking	Pre- and post-storm procurement of necessary resources; resource tracking
Planning Section	Coordination of MMS efforts; internal and external situational awareness; strategic planning	Overall coordination of hurricane planning efforts; situational awareness

a Medical Monitoring Station Shelter-in-Place Plan.

b Personal protective equipment.

c Medical Monitoring Station.

d Information technology.

e Incident Command Post.

Upon completion of the MSIPP, MMS leadership convened more than 70 key players involved in the facility’s internal and external affairs for a virtual tabletop exercise, simulating the MMS’s preparations and response to a major hurricane. The exercise ensured transparency of planning elements to the entire MMS team and encouraged identification of shortfalls or duplicative efforts. Following the exercise, a revised sector-specific plan was requested from each contributor and incorporated into a new version of the MSIPP.

## PLANNING OUTCOMES

The MSIPP serves as a consensus on how MMS operations may be adjusted to prepare for a hurricane. Major actions for each sector or agency involved fall into 1 of 6 planning topics, which are described below. Federal Emergency Management Agency (FEMA) guidelines for hurricane planning during the pandemic advise the creation of adaptable and scalable plans,^3^ and the need for this type of flexibility was evident as the MSIPP was developed and later activated for a real-world event.

### Patient Care

The MMS is not designed or equipped to provide acute care beyond stabilization and transport to a local emergency department. However, hurricane wind speeds and/or flooding are likely to interrupt EMS transport during a storm. Given the limited medical capabilities of the MMS, patient care planning focused on maximizing patient safety through reverse triage of all active patients, application of more stringent clinical criteria to all incoming patients, and expansion of critical care capabilities to serve decompensating patients when EMS transport is inaccessible.

To conduct reverse triage, the MMS clinical and medical operations teams assess all patients for high risk criteria, such as dialysis requirements, deteriorating respiratory status, or declining vital signs. The remaining patients are then reviewed on an individual basis for the potential to decompensate. All high-risk patients are evacuated to a medical special needs shelter or hospital, depending on their current status and needs. Medical special needs shelters are located in more northern regions of Louisiana and accommodate those with chronic medical needs during an evacuation.^4^

**FIGURE 1:**
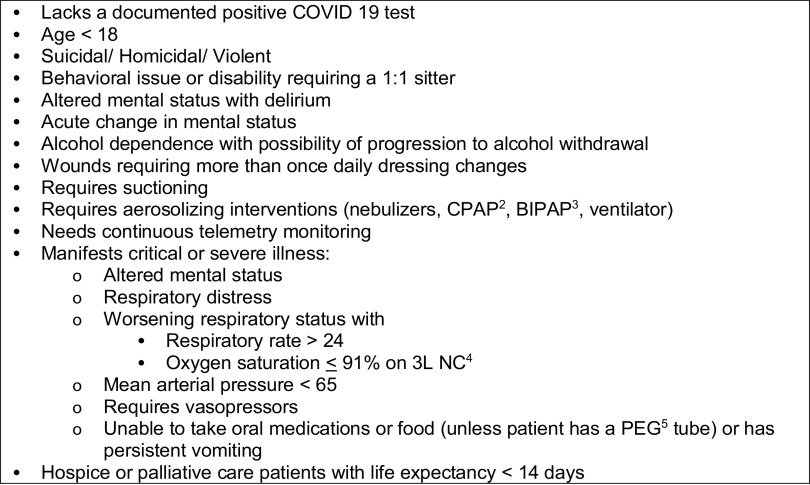
MMS^1^ Exclusion Criteria. ^1^ Medical Monitoring Station ^2^ Continuous Positive Airway Pressure ^3^ Bilevel Positive Airway Pressure ^4^ Nasal Cannula ^5^ Percutaneous Endoscopic Gastrostomy

During regular operations, the MMS accepts any patient that does not meet predefined exclusion criteria (Figure 1). However, in the event of a hurricane, all incoming patients undergo individual review by a physician before being accepted, to ensure they are appropriate for the facility in a SIP scenario. Some medical conditions typically appropriate for care at the MMS become a safety concern in the event of a hurricane where EMS transport may be significantly delayed.

Despite a more stringent patient acceptance process and evacuation/transfer of high-risk patients, there remains a potential for medical emergencies to occur during and after the storm, when EMS transport cannot safely occur. To address this, an Acute Treatment Area is activated within the MMS in the event of a hurricane. This area encompasses a cache of emergency/critical care equipment, supplies, and medications needed to resuscitate, stabilize, and maintain patients who become critical, until they can be safely evacuated. Items not typically stocked in the MMS, such as rapid sequence intubation medications, ventilators, IV pumps, and cardiac/anticoagulant/insulin infusions are available in this area. However, the use of these medications and equipment is dependent on the ability to staff the area with clinicians qualified to provide emergency/critical care. As emergency/critical care is not the focus of the MMS, this requires the procurement of additional qualified staff in the event of a hurricane.

### Interfacility Coordination Planning

In line with hospital norms across coastal Louisiana, the MSIPP is intended for use in up to a Category 3 hurricane. However, other alternative care sites and nursing homes across the state are more prone to wind damage and flooding and must evacuate for smaller storms.

The MMS is an excellent evacuation option for many COVID-19 positive patients from other alternative care sites in the state. These sites have fewer resources and stricter acceptance criteria, so most patients are expected to easily meet safe acceptance criteria for the MMS. Likewise, the MMS is a more appropriate use of resources for COVID-19 positive nursing home patients than an acute-care hospital. While the Louisiana Department of Health is assisting nursing homes in solidifying jeopardized facility evacuation plans, the MMS stands ready to accept COVID-19 positive residents that cannot be safely accommodated by a receiving facility. This will help to prevent a large influx of COVID-19 positive nursing home patients into hospitals for the sole purpose of medical sheltering.

### Wrap-Around Service Planning

Continuity of wrap-around services is critical to the ability of the MMS to remain operational. Ensuring that patients and staff continue to receive scheduled meals is essential, and without it, patient care is impossible. Therefore, the MSIPP calls for food to be stocked, stored, and cooked onsite using equipment that can function by municipal power or portable generator. The MMS’s vendors and dietician are alerted to provide special medical diets, ensuring that diabetic, cardiac, soft, and liquid diets are provided to patients who need them, even in disaster conditions. The continuation of environmental/cleaning services is also essential, especially given the infectious nature of COVID-19. To ensure dietary and environmental services continue for the duration of a hurricane and potential aftermath, plans include onsite socially distanced sleeping, hygiene, and meal accommodations for the employees who provide these services.

Other important wrap-around services, such as waste disposal, laundering, and medication delivery are likely to be interrupted and require preparation in advance of the storm. All waste is removed just before the storm and all receptacles are secured. Additional linens, towels, and gowns are prestocked with the anticipation that laundering services may be interrupted and unavailable for a period. Patient charts and medications are carefully reviewed by the clinical teams to determine the need for refills, which are procured before the landfall of any storm expected to interrupt outside pharmacy services.

### Staffing Planning

In addition to sector-specific annexes to support the MSIPP, each partner agency/section involved in the MMS was asked to submit a roster detailing the essential personnel expected to shelter at the facility through a hurricane activation. A maximum roster of 650 personnel was identified to support patient care and/or medical sheltering for up to 240 patients through a major hurricane. Given the inherent threat to staff safety, the MSIPP also accounts for the billeting and feeding of all essential personnel required to support incident command, medical care, and all necessary ancillary services. In line with guidance from the National Mass Care Strategy for Congregate Sheltering Operations in a COVID-19 Pandemic,^3^ the MSIPP provides 110 square feet of sleeping space for each staff member. Cots for staff are arranged head-to-toe, with temporary privacy curtains erected between groupings of cots to encourage social distancing. Using more than 90,000 square feet of upstairs conference and ballroom space within the MCCNO for sleeping quarters alone ensures the best-available infection control measures for staff, even when uninvolved with patient care activities. Hygienic considerations include provision of personal care items and the deployment of portable showers and toilets to the MCCNO.

### Medical Logistics Planning

Throughout the COVID-19 pandemic, health-care facilities across the nation have battled extreme shortages of critical medical supplies.^5^ While the MMS had previously identified and sourced the supplies necessary to support its operations under normal conditions, the potential need for self-sufficiency during a concomitant hurricane elevated concerns about both the variety of items required and the ability to obtain sufficient quantities during a global health crisis.

In anticipation of these challenges, the MMS developed an Emergency Inventory Package within the onsite MMS Central Supply warehouse. As a prestaged cache maintained separately from the MMS’s normal-use inventory, the package is shielded from the ebb and flow of operational inventory at the facility, thus ensuring a 14-d sustainment for over 240 patients in the event of supply chain disruption as may be expected during a high-impact tropical event. Ahead of the Atlantic-basin hurricane season, personal protective equipment (PPE) stocks at the MMS were increased from a 7-d to 14-d par level.

While the Emergency Inventory Package includes primarily those items anticipated to present difficulties for just-in-time delivery before a hurricane (ie, high-demand medical supplies), the MSIPP incorporates a list of larger resources (eg, 10 kW generators, fuel tanks, etc.) that would require dispatch to the MMS from state emergency management partners in the days before a storm. The activation of the MSIPP involves a comprehensive check of all inventory onsite 120 h before the storm’s anticipated landfall on the Louisiana coast. Given the criticality of medical supplies to the MMS mission, this timeline permits the fulfillment of “flash” resource requests from state logistics partners, adding redundancy to the Emergency Inventory Package.

**FIGURE 2:**
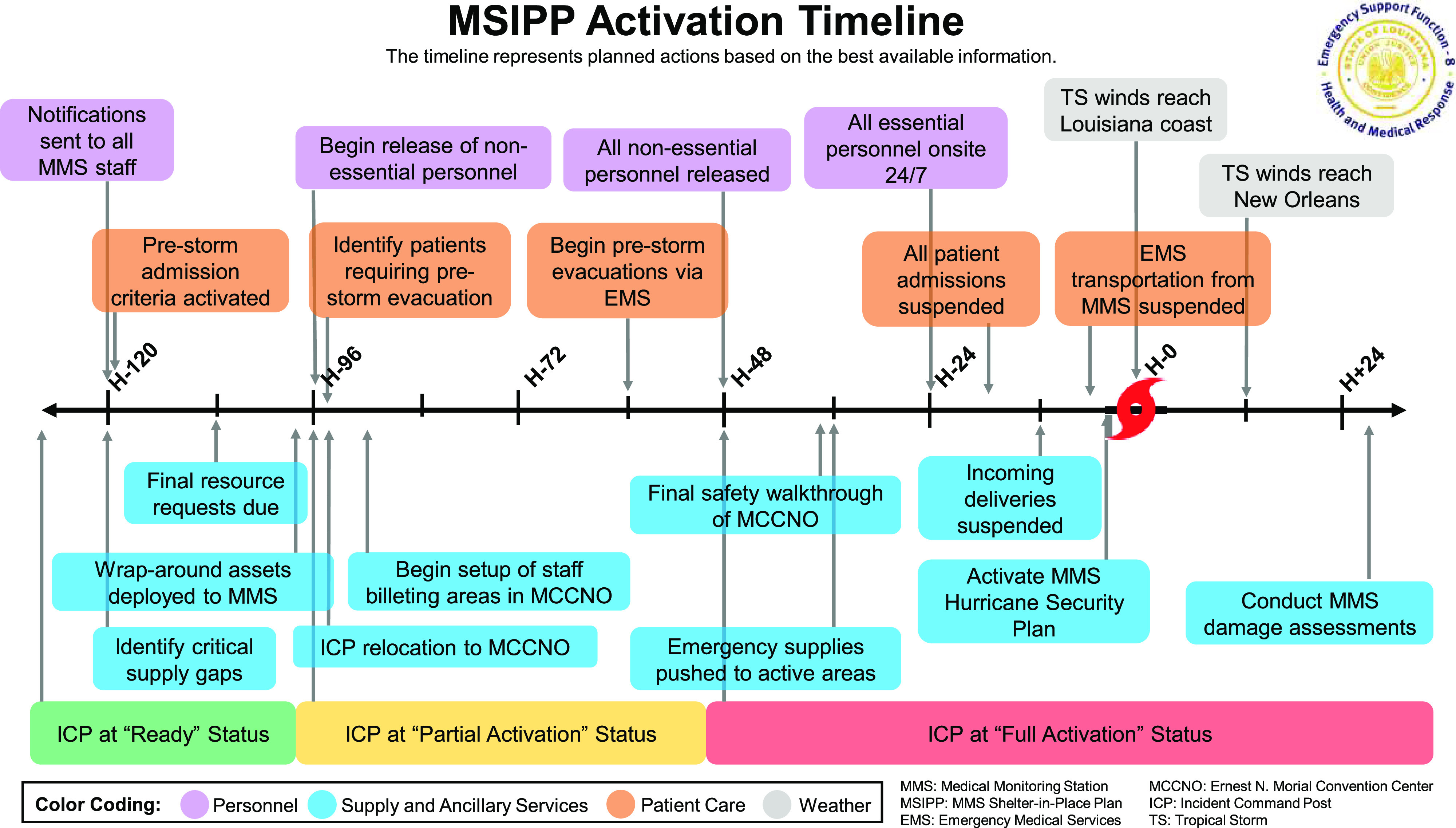
MSIPP Activation Time.

The MSIPP Activation Timeline (Figure 2) also addresses the need to suspend all incoming deliveries and outbound movements (eg, medical and standard waste) from the MMS for staff safety. Notification of vendors and staging of additional waste containment infrastructure is incorporated into the prestorm SIP timeline.

### Incident Command Post Continuity Planning

The MSIPP simultaneously addresses the continuity of the Incident Command Post (ICP), normally located in a nearby hotel. Unlike the MCCNO’s electrical redundancies and storm-hardened construction, this hotel lacks the appropriate infrastructure to ensure safety and functionality to those in the ICP during a major hurricane. Beginning 96-h before a storm’s landfall, the MSIPP calls for the relocation of the ICP to the upper floors of the MCCNO, where over 7500 square feet of meeting rooms have been earmarked for its operation. Similar to staff billeting areas, the ICP relocation footprint supports social distancing among on-duty staff.

In support of the ICP, the MSIPP also details the deployment of redundant communications equipment (700/800 MHz radios, FirstNet Long Term Evolution phones, and mobile WiFi) interoperable with the Louisiana Wireless Information System, the largest statewide radio system in the country.^6^ This staging of this communications equipment is paralleled by a deployment of 5 kW and 10 kW portable generators capable of supporting critical operations at the ICP and MMS in the event of a total power failure.

Tasked with command and control of the MMS under all circumstances, the ICP also bears the responsibility of activating the MSIPP. To clearly delineate the development of the impending hazard, the ICP adopts 3 Storm Activation Levels ahead of a tropical event: Ready Status: beginning at H-120, the ICP focus is on strategic planning and storm monitoring with state partners; Partial Activation Status: beginning at H-96, the ICP begins relocation as well as release of nonessential personnel; and Full Activation Status: beginning at H-48 and extending through the storm’s duration, the ICP and MMS are fully readied for sustainment and nonessential activities are suspended.

## DISCUSSION

On June 1, 2020, the MMS began monitoring the development of Invest 93L in the Gulf of Mexico, which would later develop into Tropical Storm Cristobal. The storm made landfall on Southeast Louisiana’s coastline early on June 7, 2020, less than 50 miles from the MMS. Originating on the same day as the onset of the 2020 Atlantic-basin hurricane season, Cristobal brought about a rapid and preliminary activation of the MMS’s evolving hurricane plans. The MMS used the planning topics discussed above to ensure continuity of operations during Tropical Storm Cristobal. Because the storm was expected to have a low impact, only select aspects of the MSIPP were activated by MMS leadership. In addition, the early date of the storm meant that not all plans were fully ready to be implemented.

### Assessment of Patient Care Planning

Many of the critical care supplies, equipment, and medications were still being acquired to stock the Acute Treatment Area. Instead of planning to use the area to treat decompensating patients during the storm, the emergency transport plan for patients was augmented. The MMS looked to outside partners to establish agreements for emergency transport in vehicles able to operate in up to 70 mph winds and/or traverse high-water conditions. Because the storm was not expected to generate winds above 70 mph, acquiring this ability for emergency evacuation was an adequate solution to an incomplete critical care cache.

In addition, the patient census was carefully reviewed by the clinical and medical operations teams before the storm, and all patients were determined to be low risk for acute decompensation. Given the ability to evacuate patients at maximum anticipated wind speeds and the lack of high-risk patients onsite, no prestorm patient evacuations were conducted. No patient decompensated or required acute care or evacuation during the storm.

### Assessment of Interfacility Coordination Planning

One local alternative care site evacuated into the MMS before the storm due to their facility’s risk of flooding. A total of 5 patients from that site were reviewed by the onsite physician before being accepted, and it was determined that all patients could safely be cared for at the MMS. Care was transitioned and transport was coordinated in advance of the storm. Patients completed their care at the MMS rather than being transferred back to the original site, to reduce disruptions in comfort and care.

### Assessment of Wrap-Around Service Planning

The MMS is located on high ground that is less prone to flooding than other areas of New Orleans, as are the vendors that supply patient meals. The MMS command team met with the food vendors to discuss the continuity of dietary operations and determined there was a low risk for interruption of this operation. Because Tropical Storm Cristobal posed only a slight risk for interruption of these services, the mobile kitchen was not deployed to the MMS. However, the MMS requested and received 30,000 shelf-stable meals ahead of the storm to support staff and patients in the event emergency food was required. These meals did not accommodate the need for special medical diets. However, no emergency meals were required, as there were no interruptions in meal services during or after the storm.

### Assessment of Staffing Planning

Each sector/agency was again asked to clarify essential personnel ahead of Tropical Storm Cristobal. Although essential personnel had already been identified in the development of the MSIPP, Cristobal was expected to be low-impact and, therefore, required a less robust activation. Although MMS leadership was prepared to billet staff inside the convention center, this was not necessary given the storm’s development. All clinical staff billeted at a hotel across the street from the convention center to avoid any transportation issues in the event of city-wide flooding.

### Assessment of Medical Logistics Planning

The most significant logistics change made for Cristobal was the suspension of all deliveries from 12 h before landfall to 6 h past the cessation of high winds. The Emergency Inventory Package was available in time for the storm, but was not opened, as supply interruptions were minimal and the operational inventory at the MMS was sufficient.

### Assessment of ICP Continuity Planning

To ensure ICP continuity, ICP leadership met regularly in the days leading up to the storm and closely monitored the situation. Essential ICP staff were identified in advance and all essential members arrived before the start of the storm and prepared to stay overnight if conditions deteriorated. A relocation of the ICP was not required for Cristobal. All communication capabilities were maintained during the event.

## CONCLUSIONS

Disaster planning in 2020 requires more originality, flexibility, and scalability than any other year in modern times. The MMS itself is a product of combined practical and creative thinking. Its function and staffing are similar to a hospital, while its temporary nature, resources, and design are similar to a shelter. Neither plans for a hospital or a shelter cover the full breadth of emergency planning needed for the facility. Shelters are classically located outside of hurricane-prone areas. Hospitals are fully equipped to manage most medical emergencies onsite. A unique plan was needed to bridge this divide.

The MMS had the unexpected opportunity to activate the MSIPP during the first week of the 2020 hurricane season. Because Tropical Storm Cristobal was a low impact event, it was not necessary or appropriate to activate all aspects of the plan. Without the opportunity to fully implement the MSIPP, the ability to wholly analyze the plan and its potential shortcomings are limited. However, this emphasized the importance of scalability and flexibility for building and implementing such plans.

Patient care planners did not rush the acquisition of critical care staff, equipment, and medications to open an Acute Treatment Area as it was not needed based on predicted wind speeds and flood impacts. This prevented an unnecessary rush to obtain assets that require careful attention to assemble and use.

Interfacility coordination with local alternative care sites requiring evacuation worked well. However, all nursing homes remained in their home facilities for the storm. Should evacuations of these sites have occurred, the MMS would have faced additional challenges due to a higher volume and acuity of patients. In addition, no prestorm transfers/evacuations were required for the MMS population, so interfacility coordination with hospitals has yet to be practiced.

The decision to continue normal operations for wrap-around services, rather than activate the MSIPP for these sectors prevented waste, as did the decision not to shelter staff onsite at the MMS. Meanwhile, identifying storm-specific essential personnel to remain onsite reduced the risk for staff traveling to and from the site. The low impact of the storm led to minimal interruptions for the logistics team and ICP, despite preparation for a more disastrous scenario.

The sustainment of a COVID-19 alternative care site through an early tropical storm presented a challenging, yet worthwhile, experience. It emphasized the need for scalability and flexibility in planning for novel combinations of hazards. By selectively activating elements of the MSIPP to meet the threat level posed by Cristobal, the MMS ensured a fiscally responsible and effective response that maximized patient and staff safety. MMS leadership continues to revise and reinforce the MSIPP to address the successes and gaps witnessed during Tropical Storm Cristobal.
